# Escin suppresses immune cell infiltration and selectively modulates Nrf2/HO-1, TNF-α/JNK, and IL-22/STAT3 signaling pathways in concanavalin A-induced autoimmune hepatitis in mice

**DOI:** 10.1007/s10787-022-01058-z

**Published:** 2022-09-05

**Authors:** Mahmoud Elshal, Sara H. Hazem

**Affiliations:** Department of Pharmacology and Toxicology, Faculty of Pharmacy, Mansoura University, ElGomhoria Street, Mansoura, 35516 Egypt

**Keywords:** Escin, Concanavalin A, CD4+, Nrf2, JNK, IL-22

## Abstract

The current study aims to investigate the possible protective effect of escin, the active constituent of a natural mixture of triterpene saponin glycoside, against immune-mediated hepatitis driven by concanavalin A (Con A) and to elucidate its possible underlying mechanisms. Adult male mice were administered Con A (15 mg/kg, intravenously) for 8 h. In the treated groups, mice were pretreated with escin daily (10 mg/kg in CMC, orally) for 4 days before Con A intoxication. In addition, escin was administered in a group to examine its effect on normal mice. Our results showed that escin inhibited Con A-induced elevation in liver enzymes (ALT, AST, and LDH) and curbed the Con A-induced hepatocyte necrosis and apoptosis together with abrogating the death pathway, JNK. Coincidentally, escin has shown a reduction in neutrophil, CD4+ T cell, and monocyte infiltration into the liver. In addition, escin modulated the cellular oxidant status by compensating for the Con A-depleted expression of the transcription factor Nrf2 and the stress protein hemeoxygenase-1. These effects were in good agreement with the restraining effect of escin on Con A-instigated overexpression of NF-κB and the pro-inflammatory cytokines TNF-α and IL-17A. Interestingly, Con A provoked the cellular protective pathway IL-22/STAT3, which was revoked by the escin pretreatment. In conclusion, escin shows extended antioxidant, anti-inflammatory, antinecrotic, and anti-apoptotic effects against Con A-induced immune-mediated hepatitis. These effects may collectively be via suppressing immune cell infiltration into the liver and selective modulation of Nrf2/HO-1, TNF-α/NF-κB, TNF-α/JNK, and IL-22/STAT3 signaling pathways.

## Introduction

Autoimmune hepatitis (AIH) is a progressive inflammation of the liver with an underestimated prevalence and unsatisfiable complete remission therapeutic output (Francque et al. 2012). Experimental discovery of new agents to manage immune-mediated hepatitis, including AIH, viral hepatitis, and iatrogenic immune-mediated hepatotoxicity, has been hindered by relatively limited animal models (Heymann et al. 2015). Concanavalin A (Con A) is a sugar-binding legume lectin that is extracted from the jack beans, *Canavalia brasiliensis*. It has a key role in drug development for human AIH, as it induces a model of acute immune-mediated hepatitis characterized by specific target organ toxicity, T cell-mediated liver disease due to its T lymphocyte mitogenic ability, and clinical resemblance (Sharma et al. 2013).

Con A-induced liver damage arises from the activation and recruitment of T lymphocytes to the liver. It was shown that after Con A intravenous injection, T lymphocytes assemble in the hepatic sinusoids and proliferate into CD4+ cells (Hazem et al. 2018). These T helper cells subsequently overproduce various inflammatory mediators causing the so-called cytokine storm. In addition, CD4+ T cells recognize the MHC of resident macrophages, resulting in overproduction of the main pro-inflammatory cytokine TNF-α (Shaker et al. 2022). Besides, Con A can induce hepatocellular apoptosis by both a caspase dependent pathway and a mitochondrial pathway (Liu et al. 2009).

Beta-escin (Escin) is the active constituent of a natural mixture of triterpene saponin glycoside derived from the horse chestnut, *Aesculus hippocastanum* (Berti et al. 1977). Escin has been shown to have multiple pharmacodynamic effects including anti-edematous, anti-inflammatory, venotonic, and free radical scavenging effects (Ottillinger and Greeske 2001). Currently, escin is therapeutically indicated for chronic venous insufficiency, varicose veins, hemorrhoids, hematoma, venous congestion, diabetic retinopathy, and postoperative edema. On the other hand, recent studies has shown its promising efficacy in multiple models of inflammation including a high fat diet (Avci et al. 2010), mouse paw edema (Matsuda et al. 1997), neuronal ischemia reperfusion injury (Gao et al. 2020), and rat pleuritis models (Yang et al. 2020). Yet, it is not fully investigated whether escin may have a protective effect on Con A-induced AIH or not, and if so, its underlying mechanisms. Accordingly, our current study aimed to investigate the possible protective effect of escin on this type of hepatitis and elucidate its possible underlying mechanisms.

## Materials and methods

### Animals

Adult male BALB/c mice (25–30 g) were supplied from Vacsera (Giza, Egypt) and maintained in a controlled setting with tap water and food ad libitum. Animal handling and experiments were reviewed and approved by the research ethics committee of Mansoura University, Mansoura, Egypt (Code number: 2022-46).

### Drugs and chemicals

Escin, Con A, *N*-methyl-2-phenylindole, 5,5′-dithiobis (2-nitrobenzoic acid), and vanadium trichloride were supplied from Sigma (St Louis, MO, USA). *N*-(1-Naphthyl)-ethylenediaminedihydrochloride, carboxymethyl cellulose (CMC), Tris, acrylamide:bisacrylamide (29:1) 40% solution, sodium dodecyl sulfate, ammonium persulfate, and *N*,*N*,*N*′,*N*′-tetramethylethylenediamine were purchased from Fisher Chemical (Leicestershire, UK). Bovine serum albumen (BSA) and Tween 20 were purchased from MP Biomedicals (Irvine, CA, USA). All other chemicals were of the highest analytical grade available.

### Experimental design

Mice were randomly divided into four groups (6 each): Group I (Control): animals were administered escin vehicle [CMC (0.5%) orally for 4 days] and Con A vehicle [normal saline (0.9%) intravenously (i.v.) in the tail vein 8h] before killing. Group II (Con A): animals received Con A (20 mg/kg) i.v. in the tail vein as 0.2% *w*/*v* solution in normal saline (0.9%) for 8h. Group III (escin): animals received escin (10 mg/kg/day) orally for 4 days as 0.1% w/v solution in 0.5% CMC in addition to Con A vehicle as described in Group I. Group IV (Escin+Con A): animals received escin, as described in group III, and Con A, as described in group II, 2h after the last escin dose.

The dose of Con A was chosen based on our preliminary studies and previous mouse studies (Trautwein et al. 1998; Streetz et al. 2001) using Con A. The escin dose was also determined based on previous studies (Du et al. 2016; Zhang et al. 2019) and our pilot study demonstrated that the 10 mg/kg dose achieved the maximum hepatoprotection without induction of liver dysfunction when administered alone.

### Sample collection and preparation

Eight hours after Con A injection, mice were anesthetized by thiopental sodium (70 mg/kg, i.p) (Elshal et al. 2021). For hepatic histological and immunohistochemical evaluations, the right median lobe was isolated and fixed in 10% (*v*/*v*) formalin in normal saline. To isolate serum samples, cardiac blood was collected via puncturing and centrifuged at 1000*g* for 10 min (at 4 ℃). Samples of liver homogenate were prepared by homogenizing a portion from the left median lobe of the liver in 20 mM Tris–HCl (containing 1 mM EDTA, pH 7.4), followed by centrifugation at 3000*g* for 20 min (at 4 ℃) and collection of supernatants. Serum and homogenate samples were stored at − 80 °C for subsequent analysis. Another portion from the left median lobe was kept and stored at − 80 °C for western blot analysis.

### Determination of liver function biomarkers

Alanine aminotransferase (ALT), aspartate aminotransferase (AST), and lactate dehydrogenase (LDH) activities were measured in serum as hepatocellular injury biomarkers by biochemical kits (Spectrum Diagnostics, Egypt).

### Determination of hepatic necroinflammation and immune cell infiltration

Fixed and paraffinized liver sections (5 μm thick) were dewaxed in xylene, mounted on slides, and stained with hematoxylin–eosin (H&E). The slides were then examined for necroinflammation and immune cell infiltration according to the criteria of the histological activity index (HAI) (Ishak et al. 1995). The scoring of necroinflammation was determined as the summation of: 0–4 for periportal or periseptal interface hepatitis; 0–6 for confluent necrosis; 0–4 for focal inflammation, focal necrosis, and apoptosis; 0–4 for portal inflammation. An average neutrophil count was determined for each group via counting ten fields with the highest aggregates of polymorphonuclear leukocytes with segmented nuclei (Elshal et al. 2019).

Additionally, immunohistochemical expression level of the neutrophil marker Ly6G in liver was evaluated via immunostaining by the avidin–biotin complex method (Guesdon et al. 1979) using anti-mouse Ly6G antibody (Cat. No: 127602; BioLegend, CA, USA) based on the manufacturer’s guidance.

Similarly, CD4+ T cell and monocyte infiltration in liver was also evaluated by immunohistochemical determination of the expression levels of the CD4+ T cells and macrophage marker F4/80 via immunostaining using mouse monoclonal CD4+ T cell antibody (M7310; Dako, Denmark) and anti-mouse F4/80 antibody (Cat. No: 123102; BioLegend, CA, USA) according to the manufacturer’s instructions. Semi-quantitative scoring of positively immunostained samples was assigned as follows: 0 for < 5%, 1+ for 6–24%, 2+ for 25–49%, 3+ for 50–74%, and 4+ for 75–100% positively stained cells (Wang et al. 1999).

### Determination of hepatic expression levels of pro-apoptotic markers

Hepatocyte apoptosis was determined by examining the pro-apoptotic markers Bax and active caspase-3 via immunostaining by the avidin–biotin complex method (Guesdon et al. 1979) using anti-Bax and anti-cleaved caspase-3 rabbit polyclonal antibodies (Cat. No: A12009; ABclonal, MA, USA and GB11532; Servicebio, Gent, Belgium, respectively) according to the manufacturer instructions.

### Determination of oxidative stress and antioxidant parameters

#### Determination of hepatic malondialdehyde (MDA) concentration

To estimate the extent of lipid peroxidation, hepatic MDA content was specifically measured using the hydrochloric acid-based medium assay previously described by Gérard-Monnier et al*.* (1998).

#### Determination of hepatic total nitrate/nitrite (NO_***x***_) content

Total nitrate/nitrite (NO_*x*_) content in the liver were determined as indicators for the nitric oxide synthesis pathway, using vanadium (III) to reduce nitrate and the Griess reaction as mentioned formerly (Miranda et al. 2001).

#### Determination of hepatic Nrf2 and HO-1 expressions

Hepatic Nrf2 and HO-1 expressions were determined immunohistochemically via the avidin–biotin complex method (Guesdon et al. 1979) using the anti-Nrf2 rabbit polyclonal antibody (Cat. No: GB113808; Servicebio, Gent, Belgium) and anti-HO-1 mouse monoclonal antibody (Cat. No: sc-136960; Santa Cruz, Texas, USA) as directed by the manufacturers.

### Determination of hepatic NF-κB expression

Hepatic NF-κB p65 expression was determined via immunostaining by the avidin–biotin complex method (Guesdon et al. 1979) using the anti-NF-κB p65 rabbit polyclonal antibody (Cat. No: bs-20159R; Bioss Antibodies, MA, USA) as directed by the manufacturer.

### Determination of TNF-α, IL-17A, and IL-22 levels

The concentrations TNF-α, IL-17A, and IL-22 were measured by the ELISA MAX™ Deluxe set (BioLegend, SanDiego, CA, USA) in the liver tissue lysates and sera. To prepare liver lysate samples, liver portions (10%) were homogenized in cold lysis buffer (10 mM Tris pH 7.4, 150 Mm NaCl, 0.5% Triton X-100) supplied with an appropriate amount of Protease Inhibitor Cocktail Set 1 (Calbiochem, USA). Thereafter, centrifugation of samples at 7000*g* for 10 min at 4 °C was carried out for isolating the supernatants to load in the 96-well plate for ELISA.

### Western blotting analysis of p-JNK1 and p-STAT3

Portions of liver weighing 20 mg were lysed in a cold buffer (50 mM Tris–HCl pH 7.6, 150 mM NaCl, 1 mM EDTA, 1% Triton X-100, 0.25% sodium deoxycholate, 0.1% sodium dodecyl sulfate) supplied with the protease inhibitor cocktail. To eliminate cell debris, samples were sonicated and incubated for 30 min on ice before being centrifuged at 10,000*g* for 20 min at 4 °C. Thereafter, the protein concentration was estimated by the Bradford assay (Miranda et al. 2001). Slab gel (10%) was applied according to Roy and Kumar (2014). After gel polymerization, equivalent sample concentrations (30 µg protein/sample) were loaded in the wells of sodium dodecyl sulfate–polyacrylamide gel and electrophoresis was done at 125 V for about 2 h. Gel was stained by 0.1% Coomassie blue R-250 for 2 h and then a solution of glacial acetic acid, methanol, and water (1:3:6, respectively) was used for distaining. Then, the separated proteins were transferred under electric current to a nylon membrane (GE Healthcare). The blotted membrane was then incubated in blocking solution for 1h at room temperature, followed by incubation overnight at 4 °C with a solution encompassing either anti-beta actin mouse monoclonal antibody (Cat. No: mAbcam8224; abcam, MA, USA), anti-JNK1 (phospho-T183) rabbit polyclonal antibody (1/1000) (Cat. No: ab47337; abcam, MA, USA), or anti-phospho-STAT3 mouse monoclonal antibody (1/1000) (Cat. No: sc-8059, Santa Cruz, Texas, USA]. After three cycles of 5 min wash, the membrane was incubated with a solution containing the appropriate concentration of the secondary antibody for 1h at 25 °C. The chemiluminescence detection was used to identify the antibody-bound protein bands, and the Totallab analysis software (Ver.1.0.1) was used for densitometry analysis (Geldoc-it, UVP, England).

### Statistical analysis

The data were analyzed using one-way analysis of variance (ANOVA) test, followed by Tukey–Kramer multiple comparison test as post hoc test and expressed as mean ± SE (*n* = 6). Kruskal–Wallis by rank and the Dunn’s multiple comparison post hoc tests were used to analyze the scores of necroinflammation and immunohistochemistry. Statistical analysis and graphing were performed by GraphPad Prism V8.01 (GraphPad Software Inc., CA, USA).

## Results

### Effects of escin on Con A-induced hepatocellular injury

**Fig. 1:**
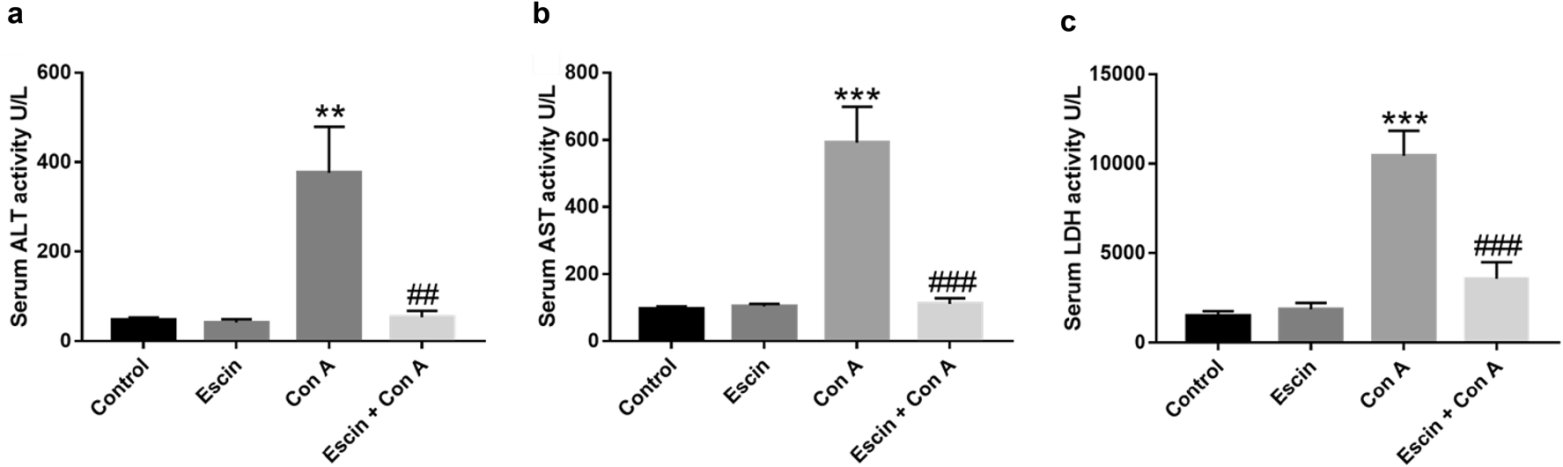
Effects of escin on serum levels of (**a**) ALT, (**b**) AST, and (**c**) LDH in Con A-injected mice. Data are expressed as mean ± SEM (*n* = 6). **p < 0.01, ***p < 0.001 compared to the control group; ^##^*p* < 0.01, ^###^*p* < 0.001 compared to the Con A group

Hepatocellular injury after 8h from Con A injection was evidenced by a significant increase in ALT (*p* < 0.01), AST (*p* < 0.001), and LDH (*p* < 0.001) serum activities in comparison with the normal mice group (Fig. 1a–c, respectively). However, administration of escin (10 mg/kg/day) for 4 days before Con A significantly decreased the peak activities of ALT (*p* < 0.01), AST (*p* < 0.001), and LDH (*p* < 0.001) compared to Con A alone. Alternatively, escin alone did not affect serum ALT, AST, or LDH activities.

### Effects of escin on Con A-induced hepatic necroinflammation and immune cell infiltration

**Fig. 2:**
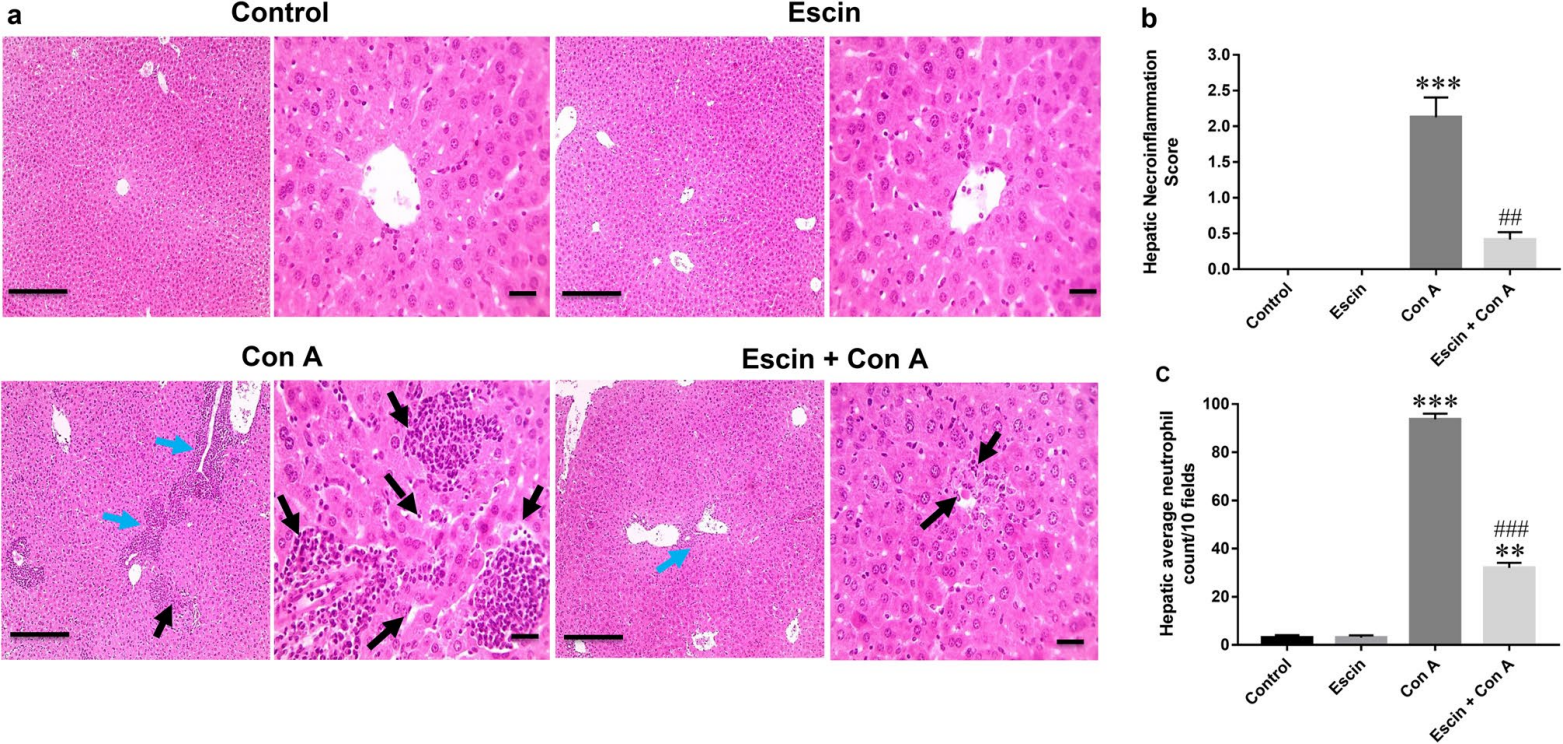
**a** Hematoxylin and eosin staining for liver sections showing normal hepatic histology in the control and escin groups, marked necroinflammatory changes characterized by severe periportal and portal lymphohistiocytic infiltration (blue arrows), heavy neutrophil aggregation (black arrows), and large area of necrosis (dashed black arrows) in the Con A group, and decreased necroinflammatory changes characterized by mild portal lymphohistiocytic infiltration (blue arrow) and very few neutrophils aggregation (black arrow), and small area of necrosis (dashed black arrow) in the treated group. Low magnification (×: 100, bar = 100 µm), High magnification (×: 400, bar = 50 µm). **b** Histopathological scoring of hepatic necroinflammation. ***p < 0.001 compared to the control group; ^##^*p* < 0.01 compared to the Con A group. **c** Histopathological assessment of average infiltrated neutrophil count/10 fields in liver. **p < 0.01, ***p < 0.001 compared to the control group; ^###^*p* < 0.001 compared to the Con A group

As shown in Fig. 2a, liver sections from the Con A group stained with H&E showed marked necroinflammatory changes characterized by severe periportal and portal lymphohistiocytic infiltration, heavy neutrophils aggregation, and large area of necrosis in comparison with the normal hepatic histology in both the control group and the group that received escin only. However, these hepatic pathological abnormalities were markedly ameliorated upon administration of escin (10 mg/kg/day) for 4 days before Con A.

These results were further illustrated by semi-quantitative scoring of necroinflammation (Fig. 2b) and counting of infiltrated neutrophils (Fig. 2c). Both the hepatic necroinflammation score and the average neutrophil count were significantly (*p* < 0.001) greater in the Con A group than in the control counterpart, and escin administration before Con A significantly (*p* < 0.01 and 0.001, respectively) reduced both of them. Alternatively, escin alone had no effect on hepatic necroinflammation score or infiltrated neutrophil count.

**Fig. 3:**
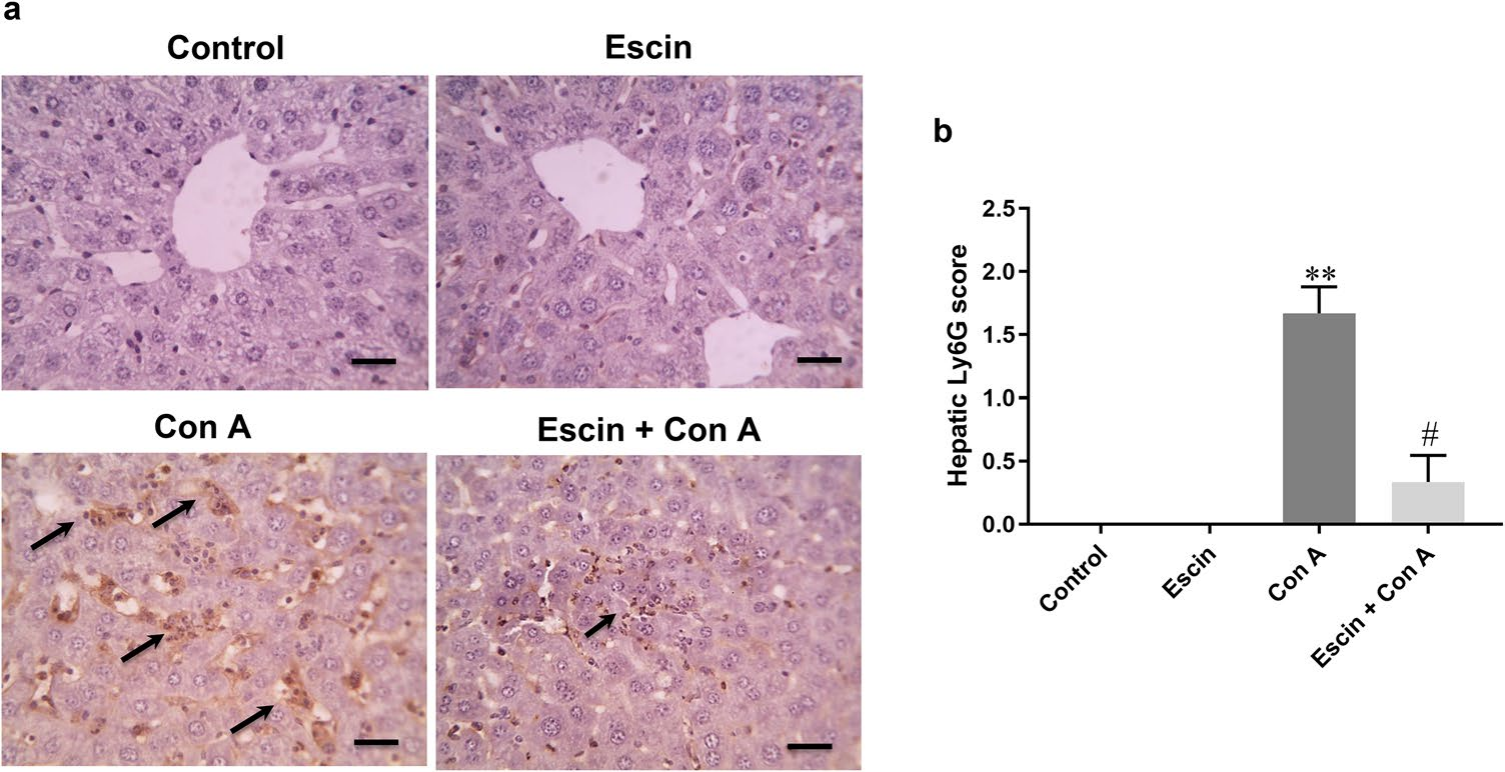
**a** Representative images of immunostained liver sections for Ly6G showing lack of staining in the control and escin groups, pronounced positive brown expression (black arrows) in the Con A group, and strong reduction of Ly6G expression (black arrow) in the treated group. IHC counterstained with hematoxylin (magnification X: 400, bar = 50). **b** Immunohistopathological scoring of hepatic Ly6G. **p < 0.01 compared to the control group; ^#^*p* < 0.05 compared to the Con A group

To confirm whether escin can limit Con A-instigated neutrophil infiltration in the liver, the hepatic expression of Ly6G positive cells was immunohistochemically assessed (Fig. 3a). Liver sections from the Con A group showed marked expression of Ly6G-positive cells that were negatively expressed in the control and escin groups. However, escin administration (10 mg/kg/day) for 4 days before Con A markedly limited Con A-induced elevation in Ly6G-positive cell. According to semi-quantitative scoring, Ly6G expression was significantly (*p* < 0.01) higher in the mice livers that received Con A alone in comparison to those of the normal mice*.* Meanwhile, hepatic Ly6G expression was significantly (*p* < 0.05) lower in the (Escin+Con A) group compared to the Con A group (Fig. 3b).

**Fig. 4:**
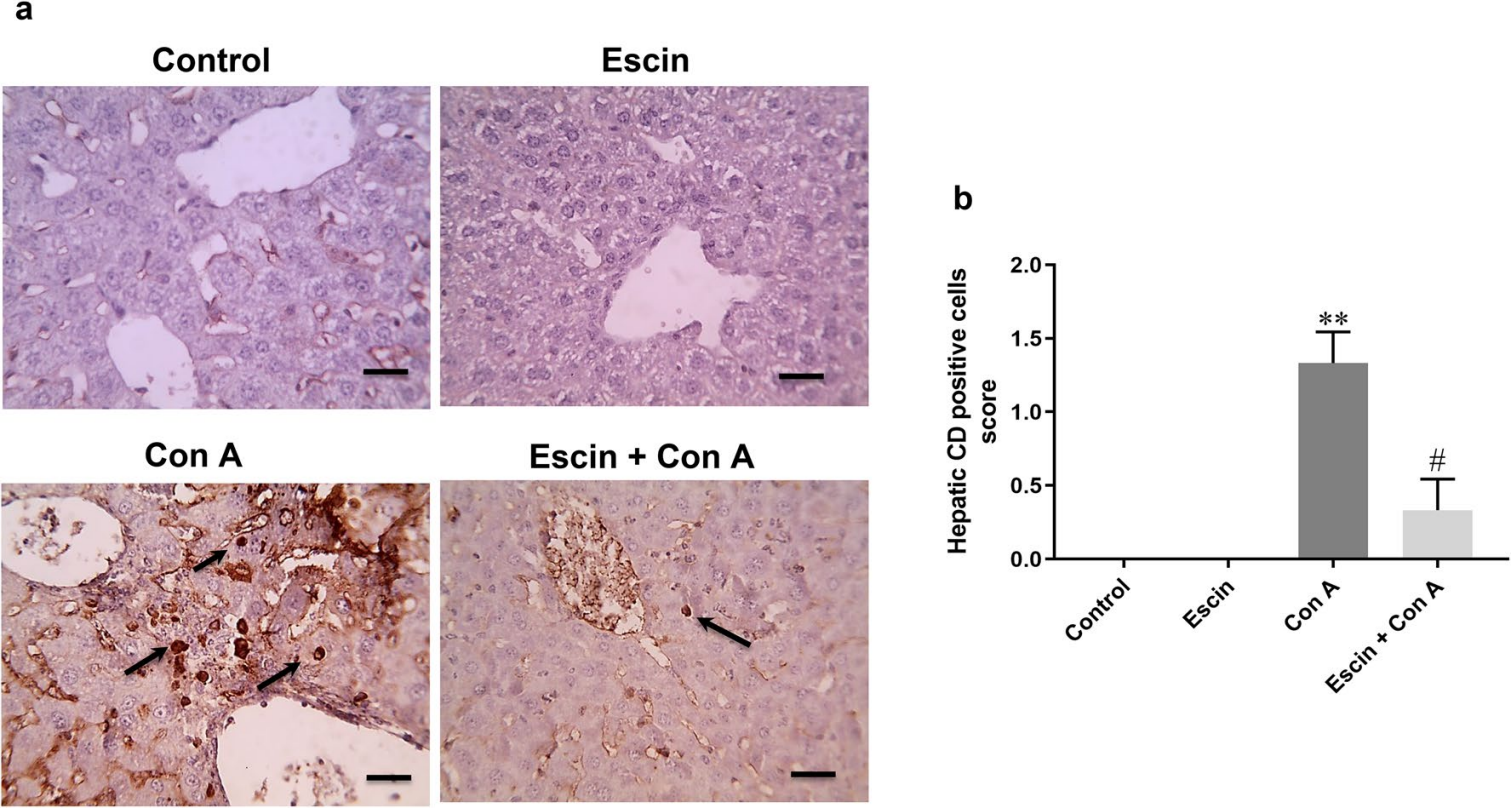
**a** Representative images of immunostained liver sections for CD4+ T cells showing nearly negative staining in the control and escin groups, markedly high number of positively stained lymphocytes in the Con A group, low number of positively stained lymphocytes in the treated group (black arrow). IHC counterstained with hematoxylin (magnification ×: 400, bar = 50). **b** Immunohistopathological scoring of infiltrated CD4+ T cells in liver; **p < 0.01 compared to the control group; ^#^*p* < 0.05 compared to the Con A group

Next, CD4+ T cell infiltration in liver was evaluated by immunohistochemical assessment of its expression (Fig. 4a). Liver sections from the Con A group showed remarkably higher percentage of CD4+ T cells positive expression than those from the control group and Escin groups. Notably, upon escin administration (10 mg/kg/day) for 4 days before Con A, infiltration of CD4+ T cells in the liver was strongly suppressed. Semi-quantitative scoring revealed that Con A-induced infiltration of CD4+ T cells was significant (*p* < 0.01) compared to the control group and that escin administration before Con A significantly (*p* < 0.05) suppressed this infiltration (Fig. 4b).

**Fig. 5:**
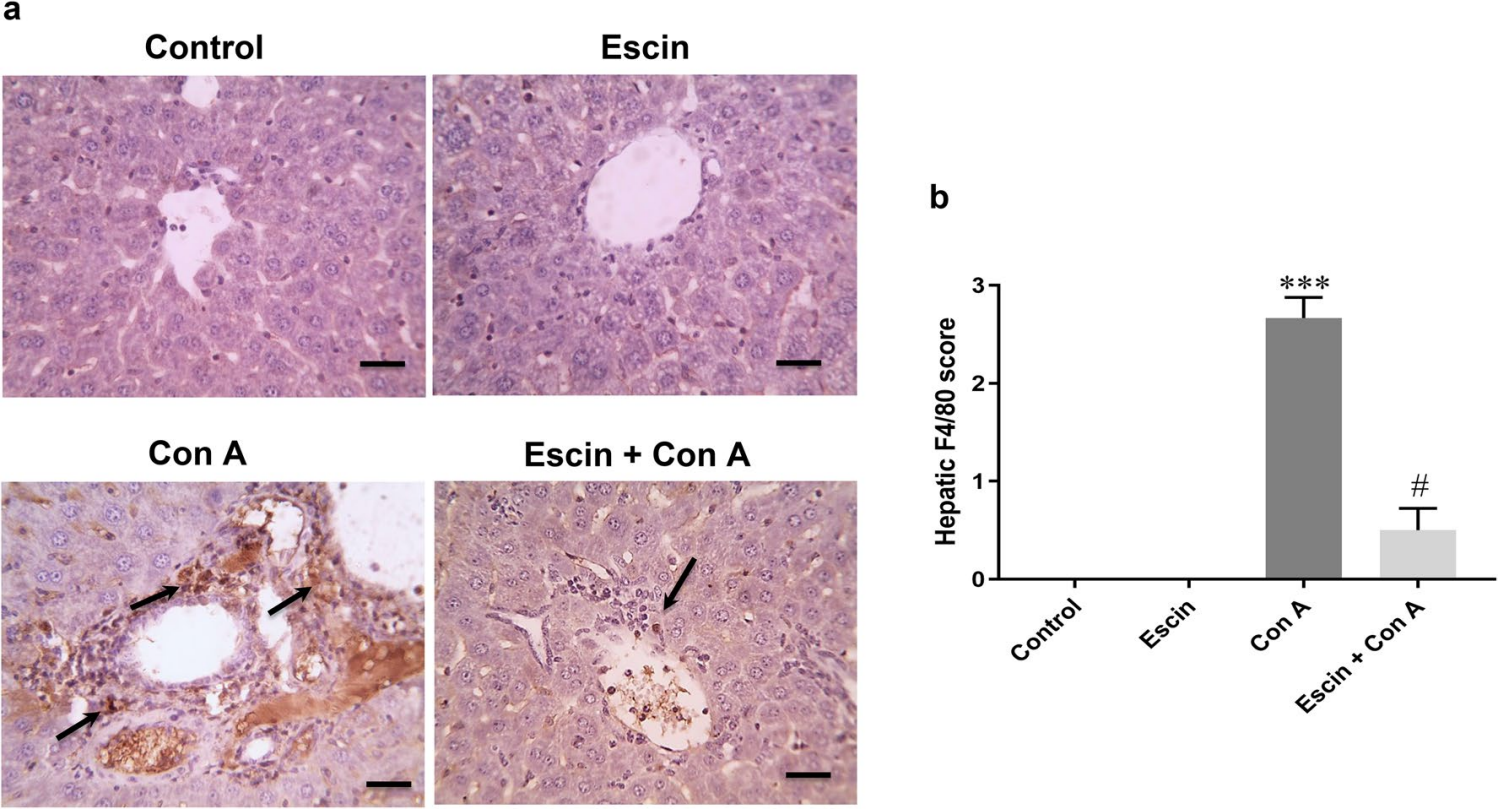
**a** Representative images of immunostained liver sections for F4/80 showing negative staining in the control and escin groups, markedly positive brown expression (black arrows) in the Con A group, and strong reduction in F4/80 expression (black arrow) in the treated group. IHC counterstained with hematoxylin (magnification ×: 400, bar = 50). **b** Immunohistopathological scoring of hepatic F4/80; ***p < 0.001 compared to the control group; ^#^*p* < 0.05 compared to the Con A group

Moreover, monocyte infiltration in the liver was evaluated by assessment of immunohistochemical expression of F4/80-positive cells (Fig. 5a). Liver sections from the control and escin groups showed negative F4/80-positive cells, while those from the Con A group showed marked expression of these cells. Conversely, escin administration (10 mg/kg/day) for 4 days before Con A strongly suppressed monocyte infiltration as well. According to semi-quantitative scoring, F4/80 expression was markedly (*p* < 0.001) elevated in the Con A group in comparison with the control counterpart*.* Interestingly, escin treatment before Con A significantly curbed (*p* < 0.05) this elevation (Fig. 5b).

### Effects of escin on Con A-induced oxidative stress in the liver

**Fig. 6:**
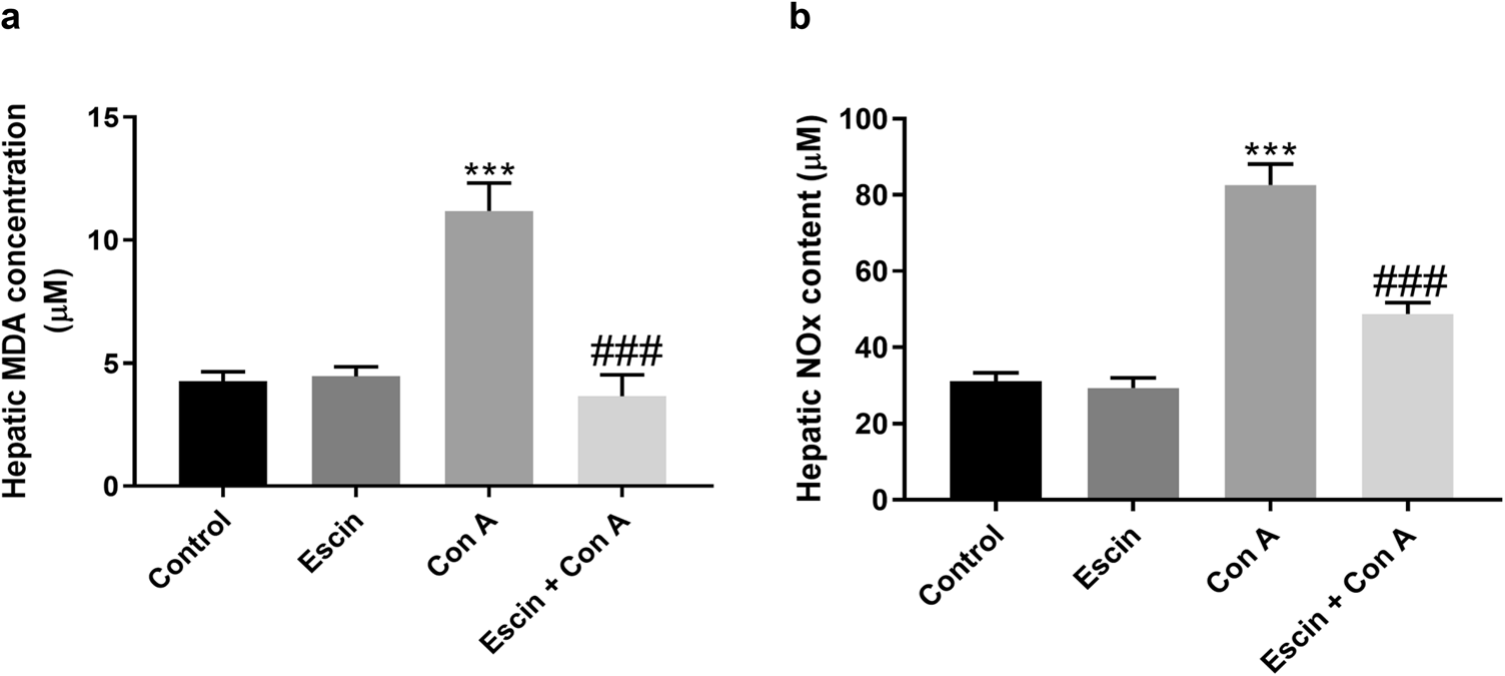
Effects of escin on liver tissue levels of (**a**) MDA and (**b**) NO_*x*_ in Con A-injected mice. Data are expressed as mean ± SEM (*n* = 6). ***p < 0.001 compared to the control group; ^###^*p* < 0.001 compared to the Con A group

Con A injection resulted in marked elevation in lipid peroxidation and nitrosative stress in liver as demonstrated by a significant (*p* < 0.001) increase in hepatic MDA and NO_*x*_ contents (Fig. 6a, b, respectively) compared to the control group. Both hepatic MDA and NO_*x*_ contents were significantly (*p* < 0.001) repressed upon the administration of escin (10 mg/kg/day) for 4 days before Con A compared to Con A alone. Alternatively, escin alone had no effect on hepatic MDA or NO_*x*_ contents compared to the control group. As all our previous results showed that using escin alone does not have any effect on the phenotype, we continued our study without the escin group.

**Fig. 7:**
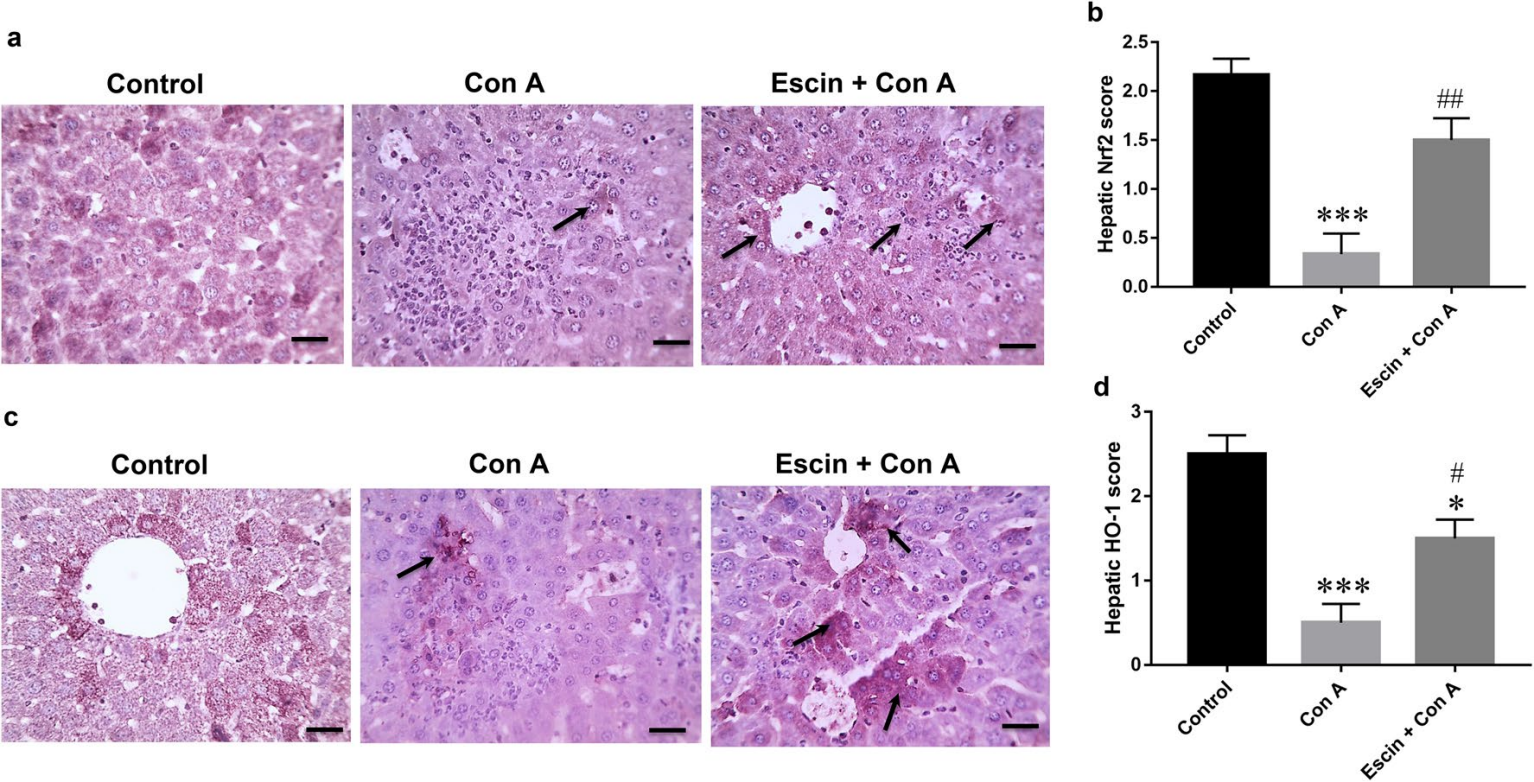
**a** Representative images of immunostained liver sections for the nuclear factor erythroid 2-related factor 2 (Nrf2) showing marked staining in the control group, ill-defined positive brown expression (black arrow) in the Con A group, and strong increase in Nrf2 expression (black arrows) in the treated group. IHC counterstained with hematoxylin (magnification ×: 400, bar = 50). **b** Immunohistopathological scoring of hepatic Nrf2. ***p < 0.001 compared to the control group; ^##^*p* < 0.01 compared to the the Con A group. **c** Representative images of immunostained liver sections for heme oxygenase-1 (HO-1) showing marked staining in control group, weak brown expression (black arrow) in the Con A group,moderate increase in HO-1 expression (black arrows) in the treated group. IHC counterstained with hematoxylin (magnification ×: 400, bar = 50). **d** Immunohistopathological scoring of hepatic HO-1. *p < 0.05, ***p < 0.001 compared to the control group; ^#^*p* < 0.05 compared to the Con A group

Trying to elucidate the underlying antioxidant mechanism of escin in this type of hepatitis, the antioxidant transcription factor Nrf2 was examined immunohistochemically. Our results demonstrated that liver sections from the control group showed marked expression of Nrf2, but the expression was very mild in the Con A group. Interestingly, pretreatment with escin markedly increased Nrf2 expression (Fig. 7a).

Next, immunostained liver sections against the antioxidant protein HO-1 were evaluated and similarly showed that HO-1 was markedly expressed in the control group, but its expression was very mild in the Con A group and upon treatment with escin, the expression was moderately increased (Fig. 7c).

Semi-quantitative scoring revealed that Con A elicited a pronounced (*p* < 0.001) reduction in the hepatic expressions of both Nrf2 and HO-1 compared to the control group, whereas escin administration (10 mg/kg/day) for 4 days before Con A caused a significant (*p* < 0.01 and 0.05, respectively) elevation in their expression (Fig. 7b, d, respectively).

### Effects of escin on Con A-induced elevation of TNF-α, IL-17A, NF-κB, and JNK1 expression

**Fig. 8:**
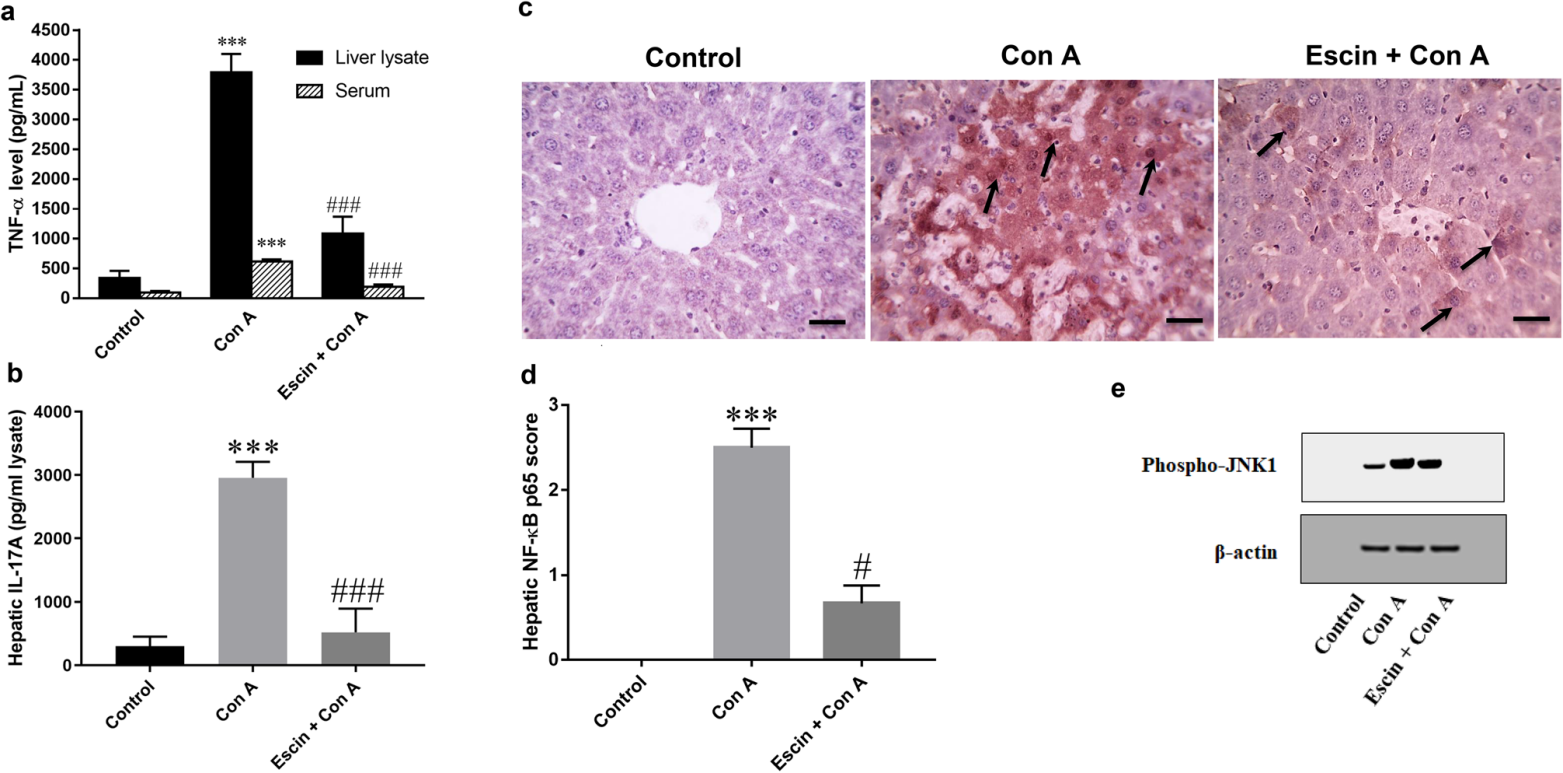
Effects of escin on **a** liver tissue and serum levels of TNF-α; **b** liver tissue level of IL-17A in Con A-injected mice. Data are expressed as mean ± SEM (*n* = 6). ***p < 0.001 compared to the control group; ^###^*p* < 0.001 compared to the Con A group. **c** Representative images of immunostained liver sections for nuclear factor kappa-B (NF-κB) p65 subunit showing weak staining in the control group, pronounced positive brown expression appears in area of damage staining nuclei and cytoplasm of hepatocytes (black arrows) in the Con A group, strong decrease in NF-κB p65 expression staining nuclei and cytoplasm of few hepatocytes (black arrows) in the treated group. IHC counterstained with hematoxylin (magnification ×: 400, bar = 50). **d** Immunohistopathological scoring of hepatic NF-κB p65. ***p < 0.001 compared to the control group; ^#^*p* < 0.05 compared to the Con A group. **e** Effect of escin on Con A-induced overexpression of phospho-JNK1

When compared to the control group, Con A injection significantly (*p* < 0.001) elevated hepatic and serum TNF-α levels and hepatic IL-17A level as well (Fig. 8a, b, respectively). Meanwhile, escin administration (10 mg/kg/day) for 4 days before Con A significantly (*p* < 0.001) reduced the elevated levels of both cytokines when compared to the Con A group.

Regarding NF-κB, immunohistochemical assessment of the transcription factor NF-κB p65 activation and translocation to the nucleus (Fig. 8c) revealed that Con A markedly elevated its expression in the cytoplasm and nucleus of hepatocytes. Alternatively, escin administration (10 mg/kg/day) for 4 days before Con A strongly reduced this expression. According to semi-quantitative scoring, Con A caused a significant (*p* < 0.001) increase in NF-κB p65 expression compared to the control group, while pretreatment with escin significantly (*p* < 0.05) reduced its expression (Fig. 8d).

Furthermore, the expression of phosphorylated JNK1 protein was evaluated by western blot. Intriguingly, the results revealed that Con A injection substantially elevated the expression of phosphorylated JNK1 protein in the liver. However, escin administration (10 mg/kg/day) for 4 days before Con A limited this elevation (Fig. 8e).

### Effects of escin on Con A-induced elevation of pro-apoptotic markers

**Fig. 9:**
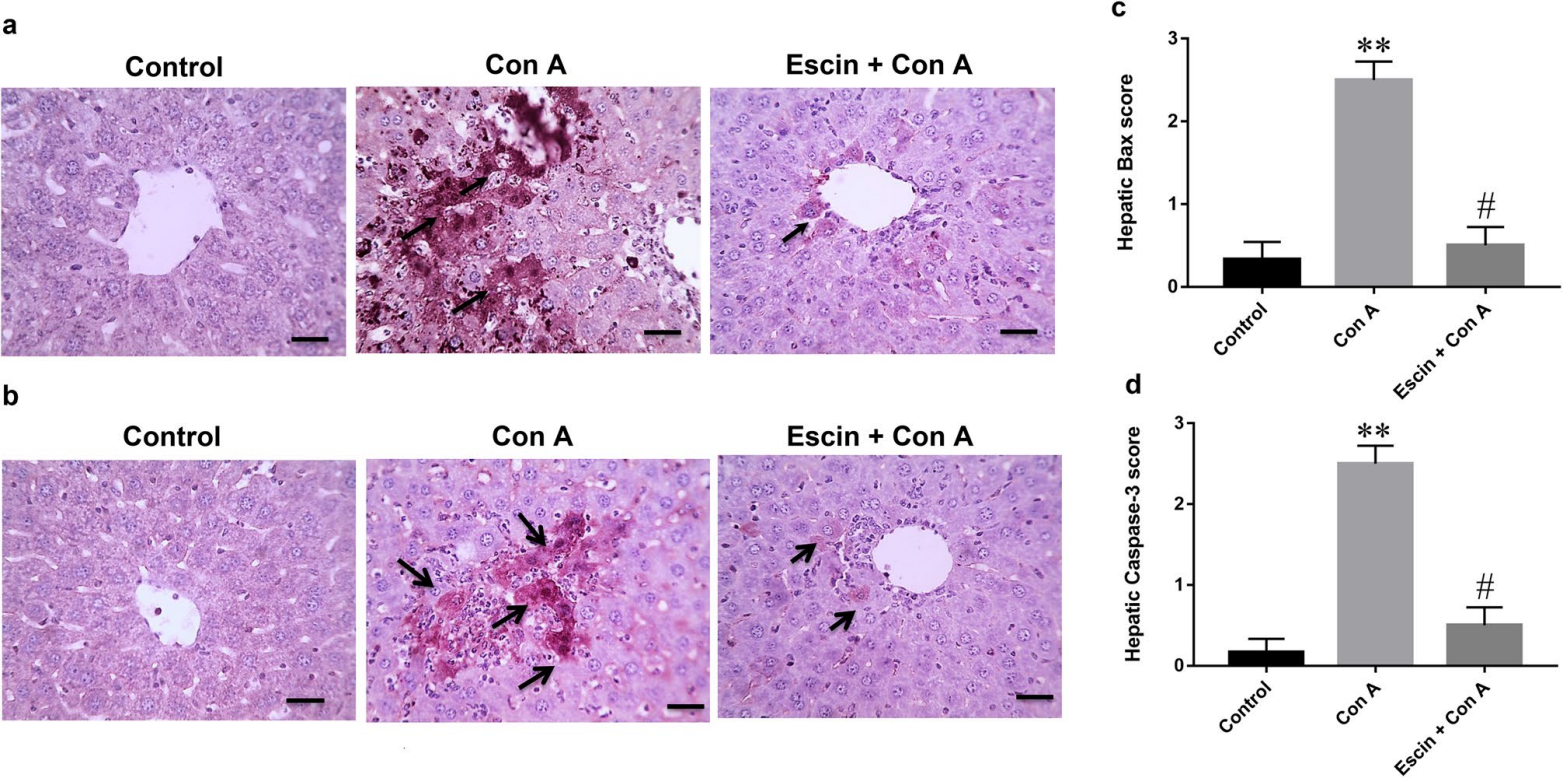
**a** Representative images of immunostained liver sections for Bax. Cells showing ill-defined staining in the control group, pronounced positive brown expression (black arrows) in the Con A group, and marked decrease in Bax expression (black arrow) in the treated group. IHC counterstained with hematoxylin (magnification ×: 400, bar = 50). **b** Immunohistopathological scoring of hepatic Bax. **p < 0.01 compared to the control group; #*p* < 0.05 compared to the Con A group. **c** Representative images of immunostained liver sections for cleaved caspase-3 showing negative staining in the control group, higher positive brown expression (black arrows) in the Con A group, and marked decrease in cleaved caspase-3 expression (black arrows) in treated group. Immunohistochemistry counterstained with hematoxylin (magnification ×: 400, bar = 50). **d** Immunohistopathological scoring of hepatic cleaved caspase-3. **p < 0.01 compared to the control group; #*p* < 0.05 compared to the Con A group

Regarding pro-apoptotic markers, immunostained liver sections against the pro-apoptotic protein Bax (Fig. 9a) showed negative expression in the control group; however, marked expression appeared in the Con A group. Obviously, this expression was markedly decreased in the treated group.

Likewise, immunostained liver sections against the apoptotic executioner caspase-3 (Fig. 9c) showed negative expression in the control group; however, marked expression appeared in the Con A group. Obviously, this expression was markedly decreased in the treated group.

Semi-quantitative scoring revealed that expressions of the pro-apoptotic proteins Bax and caspase-3 (Fig. 9b, d, respectively) in the livers of the Con A-challenged mice were significantly (*p* < 0.01) elevated when compared with those of normal mice, but was significantly (*p* < 0.05) reduced upon pretreatment with escin (10 mg/kg/day) for 4 days. Thus, Con A-induced apoptosis was effectively ameliorated by escin.

### Effects of escin on Con A-induced elevation of IL-22 level and STAT3 activation

**Fig. 10:**
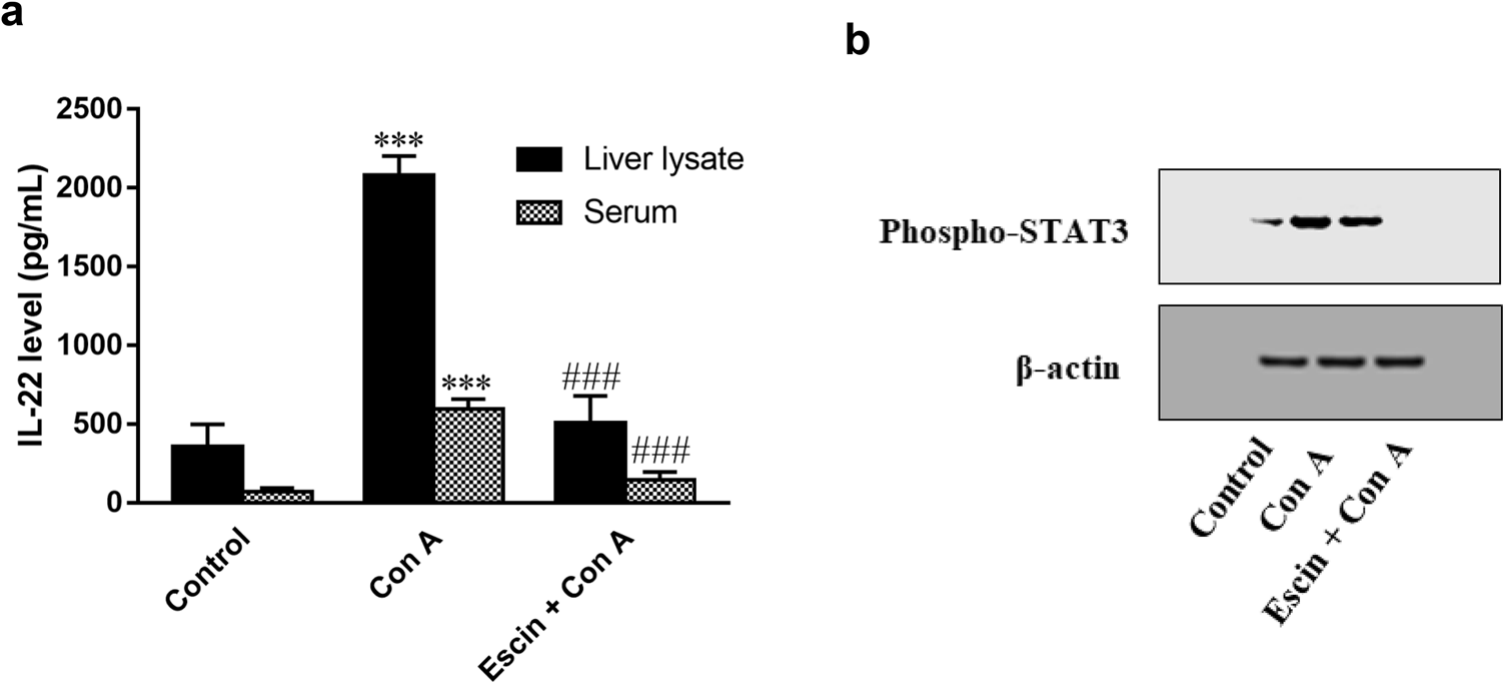
**a** Effects of escin on liver tissue and serum levels of IL-22 in Con A-injected mice. Data are expressed as mean ± SEM (*n* = 6). ***p < 0.001 compared to the control group; ^###^*p* < 0.001 compared to the Con A group. **b** Effect of escin on Con A-induced overexpression of phospho-STAT3

Interestingly, hepatic and serum levels of IL-22 were significantly (*p* < 0.001) elevated by Con A injection compared to the control group. However, these levels were significantly (*p* < 0.001) reduced upon escin administration (10 mg/kg/day) for 4 days before Con A in comparison to the group that received Con A alone (Fig. 10a).

Next, the phosphorylated STAT3 protein was evaluated by western blot. The results revealed that Con A injection strongly elevated phosphorylated STAT3 protein expression; however, this elevated expression was diminished upon treatment with escin (10 mg/kg/day) for 4 days before Con A (Fig. 10b).

## Discussion

The current standard treatment regimens for autoimmune hepatitis are based on immune system suppression (Czaja 2016) with an outcome of relapse in 50–85% of adults and 60–80% of children after treatment withdrawal (Mieli-Vergani et al. 2018), treatment failure in 9% of patients, and incomplete response in 13% of patients (Manns et al. 2010). Such data encouraged us to dig for new strategies in the management of immune-mediated hepatitis to increase the complete remission rates.

Accordingly, the main objective of our study was directed at examining the possible protective effect of escin against autoimmune hepatitis, with elucidation of the main targeted signaling pathways underlying this type of hepatitis using Con A-induced hepatitis mouse model. The results showed a significant elevation of ALT, AST, and LDH in the Con A group compared to the control group denoting liver cell injury with concomitant release of liver enzymes into the circulation. Concordantly, the histopathological examination of the H&E hepatic sections showed normal histological findings in the control group unlike the Con A group that showed marked necroinflammatory changes. The mice group that was administered escin only showed normal serum liver enzymes and normal histology denoting that escin does not possess hepatotoxic nature. On the other hand, escin pretreated group normalized the pathological changes induced by Con A.

Immune cell infiltration is substantial in the development of liver injury not only by producing pro-inflammatory mediators, but also via the recruitment of other leukocytes to the hepatic tissues, and thereby augmenting the inflammatory response (Adams et al. 2010). Neutrophils arrive at the liver as soon as 4h after Con A administration, representing the first leukocyte type to arrive at inflammation sites and, therefore, form the first line of immune defense (Bonder et al. 2004).

Our results showed that Con A intoxication resulted in heavy neutrophil aggregation in the hepatic tissue as demonstrated by histopathological examination of the H&E stain. This was also confirmed by the immunohistochemical investigation of the neutrophil marker Ly6G that showed marked expression in the hepatic sinusoids. In addition, the nitrosative stress was significantly elevated in the Con A group which can be attributed to the hepatic infiltration with neutrophils that highly express the inducible nitric oxide synthetase. Pretreating the mice with escin for 4 days was sufficient to suppress hepatic infiltration with neutrophils, as manifested by the reduction of the neutrophil infiltration, Ly6G expression and hepatic content of NO_*x*_.

It has been previously reported that CD4+ T lymphocytes are the paramount stimulator of Con A-induced liver injury. Adams et al. 2010 had confirmed this by canceling the effect of CD4+ T cells, where i.v. injection of Con A was not able to develop leukocyte recruitment and hepatic inflammation (Adams et al. 2010). Neutrophils interpose inflammatory response via recruitment other leucocytes including CD4+ T cells, where depleting neutrophils by anti-mouse antibody directed against Ly6G resulted in a marked attenuation of Con A-instigated CD4+ T lymphocyte recruitment to the liver (Tiegs et al. 1992). Moreover, neutrophils orchestrate recruitment and differentiation of macrophages (Selders et al. 2017).

These findings were in a good agreement with our results, where the immunohistochemical analysis of CD4+ T cells and the macrophage marker F4/80 showed strong positive expression in the lectin-mediated injury group, which can be linked in part to the observed elevated neutrophil infiltration. However, the intense expression was reduced in a significant way in the escin pretreated groups. Therefore, escin not only suppressed hepatic neutrophil infiltration, but also suppressed CD4+ T cell and monocyte recruitment into the liver as well.

It has been found that escin boosts endogenous antioxidant capacity via scavenging free radicals and retards oxidative degradation of lipids (Vašková et al. 2015; Jiang et al. 2011). This was shown by the reduction in the lipid peroxidation product MDA in the pretreated escin before Con A-challenge. In the same context, NO_*x*_ of the liver assessment exhibited a marked elevation in the Con A group which was substantially lowered in the escin group. Escin directly trap NO and hinder its interaction with O_2_ or ${\text{O}}_{{2}}^{ \cdot - }$, thereby preventing the generation of RNOS including the highly reactive peroxynitrite (ON${\text{O}}_{{2}}^{ \cdot - }$) (Vašková et al. 2015).

Furthermore, escin prior to Con A challenge boosted the cellular antioxidant defense mechanism as shown by the elevated expression of the Nrf2 and HO-1. Nrf2 enhances cellular antioxidant capacity by increasing the expression of genes whose protein products detoxify and eliminate ROS (Ma 2013). Moreover, Nrf2 is a positive transcription activator of HO-1 which is an antioxidant cellular defense enzyme (Sun et al. 2002).

Collectively, escin normalized cellular oxidant status by controlling ROS and RNS levels along with boosting the cellular antioxidant capacity. Thereby, this will interfere with the inflammatory signal transduction pathways including the TNF-α pro-inflammatory and pro-apoptotic signaling cascades. The pro-inflammatory cytokine TNF-α is chiefly produced from the activated macrophages and T lymphocytes which are highly activated in the Con A model (Balkwill 2009). TNF-α engagement to TNFR1 is involved in activating downstream effectors and mediating programmed cell death signaling via NF-κB and stress-mediated protein kinases, JNK 1 signaling (Wajant et al. 2003).

The pro-inflammatory downstream effectors of NF-κB include activation of naïve CD4+ T cells and their differentiation into Th-17 cells which are major producers of the pro-inflammatory cytokine IL-17A, which in turn mediates monocyte and neutrophil recruitment to the site of inflammation (Liu et al. 2017). TNF-α activates the death signaling pathway, JNK 1 that turns on the apoptotic machinery by activation of pro-apoptotic genes with the expression of death factors such as the Bax protein. In addition, JNK turns off the cell survival pathway. Finally, JNK relieves the inhibition on caspase-8 to initiate the caspase-dependent apoptosis (Weston and Davis 2007).

This vicious cycle of inflammation has been investigated in our study to elucidate the underlying mechanistic signaling of escin. The expression of the pro-inflammatory TNF-α in both serum and liver tissue was significantly elevated in the Con A group compared to the normal control, denoting the beginning of the massive inflammatory signaling in response to tissue damage. As was foreseeable, the downstream effectors of TNF-α was statistically elevated in the Con A group, where NF-κB expression was evident in both the nucleus and the cytoplasm as well as IL-17A expression in the hepatic tissue, compared to the normal control. Furthermore, western blotting of the stress-activated protein kinase JNK revealed increased expression in the lectin-induced injury group that was concordant with the apoptotic indices Bax and caspase-3. Interestingly, all these pathological inflammatory markers were crippled in a significant manner in the escin pretreated group.

Although activated T cells play a well-documented role in promoting the inflammatory response, they also have a hepatoprotective role to limit the inflammatory response; otherwise the inflammation would be fatal. Evidence suggests that activated T cells produce anti-inflammatory and anti-apoptotic cytokines, among which is IL-22. This member of the IL-10 family acts as a cellular protective cytokine in liver injury, especially those mediated by T cells, by inducing multiple downstream effectors including STAT3 (Pickert et al. 2009). In our study, the expression of IL-22 in both liver tissue and serum was significantly elevated in the Con A group where the T lymphocytes were simultaneously activated, confirmed by CD4+ T cell expression. However, in the escin pretreated group, where the T cells activation has been interrupted, the IL-22 expression was reduced compared to the Con A group. At the same time, western blotting of the STAT3 showed consistent results, where STAT3 expression was escalated in the Con A group, but abrogated in the Con A group pretreated with escin.

## Conclusion

**Fig. 11:**
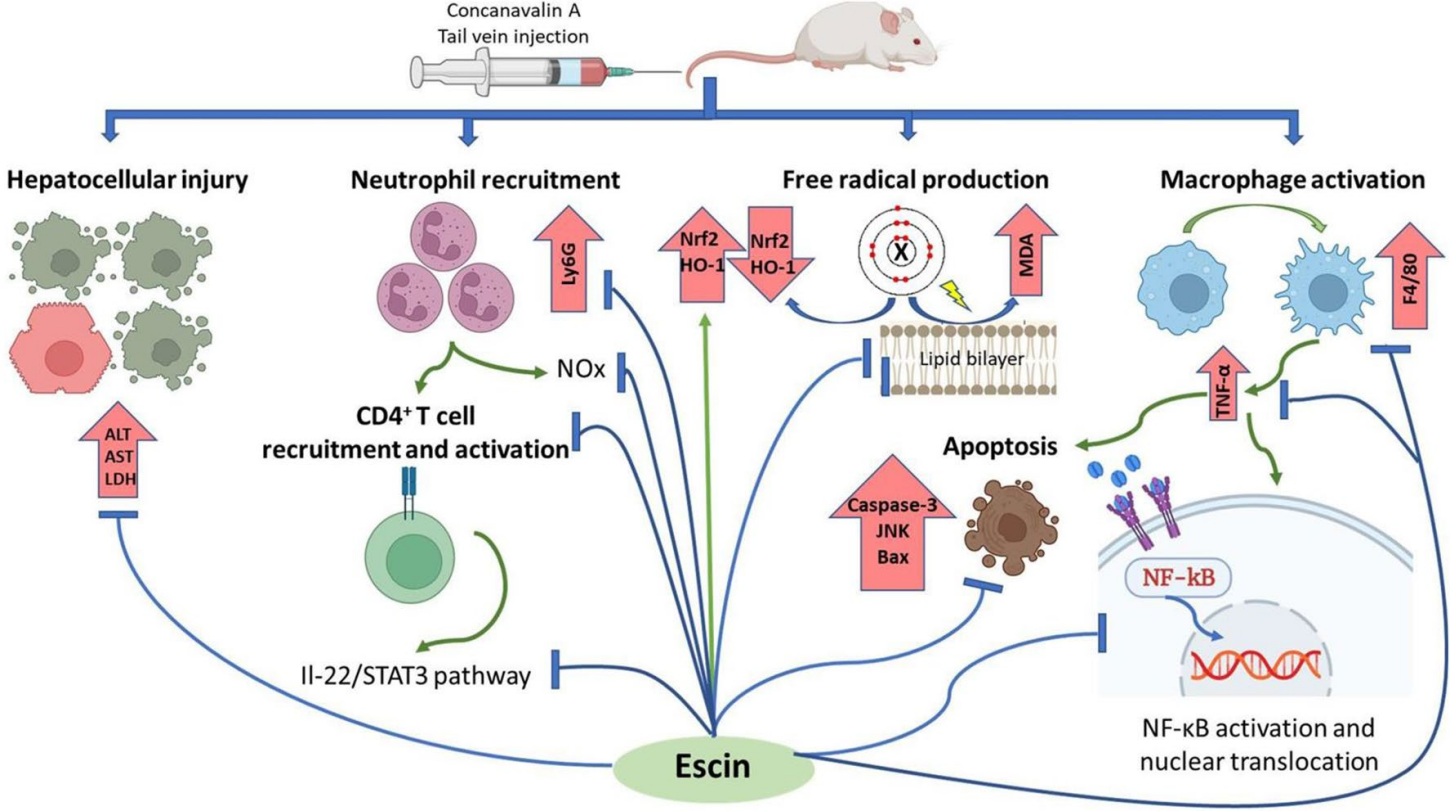
Pictorial scheme summarizing the proposed underlying mechanisms of the hepatoprotective effect of escin against Con A-induced autoimmune hepatitis in mice

Escin could protect against Con A-induced autoimmune hepatitis in mice via suppressing infiltration of neutrophils, CD4+ T cells, and monocytes into the liver, activation of Nrf2/HO-1 signaling pathway, inhibiting TNF-α/NF-κB, TNF-α/JNK, and IL-22/STAT3 signaling pathways. These characteristic and selective modulatory effects of escin lead to its extended antioxidant, anti-inflammatory, antinecrotic, and anti-apoptotic effects against this type of hepatitis (Fig. 11). Accordingly, we look forward to clinical trials to repurpose this promising natural agent as an effective, safe, and economical therapeutic option for controlling T cell-mediated hepatic disorders.

## Data Availability

The datasets generated during and/or analyzed during the current study are available from the corresponding author on reasonable request.
